# GRAVES’ DISEASE IS ASSOCIATED WITH INCREASED RISK OF CLINICAL ALZHEIMER’S DISEASE

**DOI:** 10.1093/geroni/igad104.1896

**Published:** 2023-12-21

**Authors:** Arseniy Yashkin, Stanislav Kolpakov Nikitin, Galina Gorbunova, Julia Kravchenko, Anatoliy Yashin, Igor Akushevich

**Affiliations:** Duke University, Durham, North Carolina, United States; Duke University, Durham, North Carolina, United States; Duke University, Durham, North Carolina, United States; Duke University School of Medicine, Durham, North Carolina, United States; Social Science Research Institute, Duke University, Durham, North Carolina, United States; Duke University, Durham, North Carolina, United States

## Abstract

Identification of modifiable risk factors for Alzheimer’s Disease (AD) onset is an important aspect of controlling the burden imposed by this disease on an increasing number of older U.S. adults. Graves’ disease (GD), the most common cause of hyperthyroidism in the U.S., has been hypothesized to be associated with increased AD risk, but there is no consensus. In this study, we explore the link between GD and risk of clinical AD. Cox and Fine-Grey models were applied to a retrospective propensity-score-matched cohort of 15,505 individuals with GD drawn from a nationally representative 5% sample of U.S. Medicare beneficiaries age 65+ over the 1991-2017 period. Results showed that the presence of GD was associated with a higher risk of AD (Hazard Ratio [HR]:1.15; 95% Confidence Interval [CI]:1.07-1.23). Magnitude of associated risk varied across subgroups: Males (HR:1.19; CI:1.01-1.41), Females (HR:1.09; CI:1.02-1.18), Whites (HR:1.13; CI:1.04-1.20), Blacks (HR:1.33; CI:1.04-1.20). Competing risk estimates were consistent with these findings. A potential mechanism connecting GD and AD may involve shared etiological factors between the two diseases. Although replication of our findings is needed, they suggest that GD prevention and treatment may contribute to reducing the burden of AD in U.S. older adults.

